# Scopoline Not Hyoscine

**Published:** 1907-06

**Authors:** 


### Scopoline Not Hyoscine.

A CAUTION!

In the *Archiv fuer Gynaekologie* Steffen gives same interesting details as to the use of scopolamine-morphine by Leopold. The latter has employed this method in three hundred labor cases. His verdict is that the method does not accomplish the desired results, it can not be regarded as harmless for mother and child, and in private practice the by-effects liable to develop may render medical aid requisite at any moment. When men come to conclusions so opposite as those of Leopold and those reported by Gauss, we, to whom each observer is equally trustworthy and free from bias, can only attribute the diversity to a difference in technic. That this is so may be seen by Gauss' examination of Hocheisen's method. Gauss secured a specimen of the solutions employed by Hocheisen and tried them in ten cases, the results being far worse than those reported by Hocheisen. Every objection raised by Leopold has been examined and disproved by Gauss in his much larger experience. Weakness of the labor pains did not occur to any material extent, more frequently or more markedly than in cases where this anesthetic was not used, nor were version and forceps required with greater frequency. The vomiting could only have been accidental, since it did not occur in Gauss' cases, excepting when it had commenced before the anesthetic was given. So also as to the perils to the child; Gauss showed that the mortalities of both mother and child were much less than they had been before this anesthetic was employed.

The extract, as presented in the *Journal of the American Medical Association*, gives palpable evidence of anxiety to make out a case against this anesthetic method. Even Gauss is made to rank as an objector to the method, by quoting eight troublesome cases which occurred, out of his one thousand; just as if such things never happened unless scopolamine was employed. To any one who wants the whole truth, and not a garbled *ex parte* statement, we refer to Gauss' statistics as given by Holt, in the May number of the *American Journal of Clinical Medicine*. But even were the account given a fair one, the reader will note that it nevertheless relates to the use of scopolamine, which, as commercially presented, *is not the same thing as the hyoscine used in America.* It is much as if men should insist that, because Germans injure themselves drinking too much beer, we in America should abstain from coffee.

The above being the gist of our knowledge of this subject to date, and the therapeutic difference between hyoscine, a true alkaloid, and scopolamine (or so-called hyoscine from scopola—a serious error of nomenclature) a mixed, uncertain product, being well established in favor of hyoscine, we caution our readers who are interested (and all should be) to use only H-M-C Abbott hyoscine, morphine and cactin comp.), the original American product and one which, like all the Abbott line, may be depended upon.—*Advance Sheets, Editorial in the American Journal of Clinical Medicine for July.*

